# Targeting the IL34-CSF1R axis improves metastatic renal cell carcinoma therapy outcome via immune-vascular crosstalk regulation

**DOI:** 10.1016/j.isci.2025.112752

**Published:** 2025-05-26

**Authors:** Andrea Emanuelli, Wilfried Souleyreau, Tiffanie Chouleur, Bram Boeckx, Yasmine Pobiedonoscew, Lindsay Cooley, Marie-Alix Derieppe, Julie Martineau, Damien Ambrosetti, Jean-Christophe Bernhard, Catherine M. Sawai, Diether Lambrechts, Thomas Mathivet, Andreas Bikfalvi

**Affiliations:** 1 University of Bordeaux, INSERM, U1312 BRIC, Tumor and Vascular Biology Laboratory, Pessac, France; 2 Laboratory of Translational Genetics, Department of Human Genetics, VIB-KU Leuven, 3000 Leuven, Belgium; 3 VIB Center for Cancer Biology, 3000 Leuven, Belgium; 4 University of Bordeaux, INSERM, U1312 BRIC, Modeling Transformation and Resistance in Leukemia Laboratory, Bordeaux, France; 5 Animalerie Mutualisée, Service Commun des Animaleries, Université de Bordeaux, 33000 Bordeaux, France; 6 Department of Pathology, University Côte d'Azur, CHU Nice, Nice, France; 7 Urology Department, Bordeaux University Hospital, Bordeaux, France

**Keywords:** Molecular biology, Cancer

## Abstract

Current therapies ultimately fail to eradicate metastatic renal cell carcinoma (RCC). Validated biomarkers and a better understanding of the mechanisms causing therapy resistance are still needed. Here we demonstrate that interleukin-34 (IL34) is associated with poor prognosis, metastasis, and therapy resistance in RCC. In mice, single-nucleus RNA sequencing and phenotyping reveal that the IL34-enriched tumor microenvironment displays immunosuppression and nonfunctional vasculature, two key features of therapy resistance. Mechanistically, IL34 increases migration of monocyte-derived tumor-associated macrophages (MD-TAMs) in primary tumors and lung metastases through colony-stimulating factor 1 receptor (CSF1R). Blockade of CSF1R by the Food and Drug Administration-approved drug pexidartinib contrasts MD-TAMs accumulation observed in the IL34-enriched microenvironment and improves response to sunitinib or anti-PD1 treatment to reduce metastatic growth. Altogether, our data highlight the role of the IL34-CSF1R axis in regulating the tumor immune-vascular crosstalk in RCC and indicate pexidartinib as a therapeutic alternative in combination with current therapies.

## Highlights

• IL34 expression positively correlates with progression of RCC in patients
• IL34-CSF1R axis drives the migration of MD-TAMs in the TME
• IL34-controlled MD-TAMs drive RCC via the tumor immune-vascular crosstalk regulation
• CSF1R inhibitor pexidartinib blocks the protumor activities of the IL34-CSF1R axis

## Graphical abstract

**Figure:**
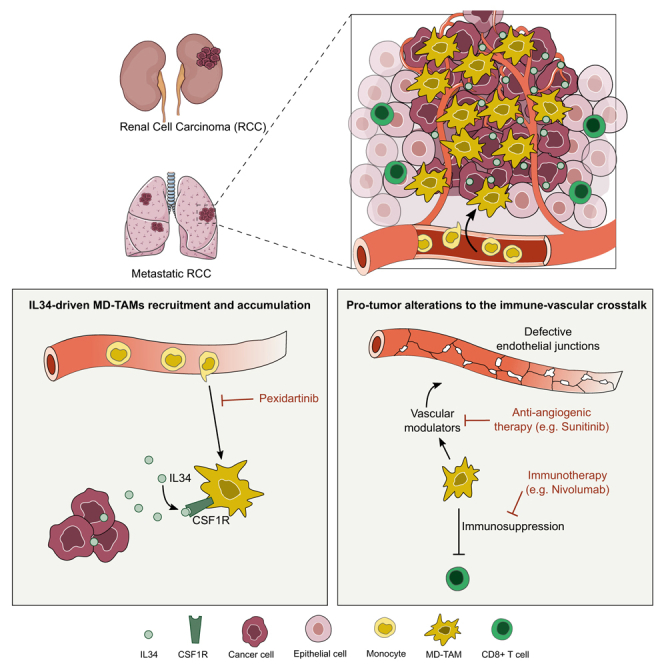

## Introduction

Kidney cancer accounts for approximately 156,000 deaths worldwide annually, and its mortality rate is projected to double by 2050.^1^ Renal cell carcinoma (RCC) represents approximately 85%–90% of kidney cancers, and surgery is the first-line treatment for primary RCC. However, metastatic RCC is diagnosed in one-third of patients and has a low five-year survival rate—close to 10%.^2^^,^^3^ Current RCC therapies target two main features of the tumor microenvironment (TME): the immune system (e.g., immune checkpoint inhibitors) and the tumor vasculature (e.g., blockers of the vascular endothelial growth factor (VEGF)/VEGF receptor(VEGFR) axis).^4^^,^^5^ However, such therapies are rarely curative, and drug resistance is almost inevitable. Thus, more effective therapeutic approaches to eradicate RCC are urgently needed. The scarcity of knowledge about the pathological mechanisms driving progression and the lack of validated targetable biomarkers are the two main reasons for treatment failure.^6^

In our previous study, we investigated the transcriptomic changes in renal cancer cells during tumor progression in a murine RCC model and identified interleukin-34 (IL34) as a potential driver.^7^ IL34 regulates the development of mononuclear phagocytic cells in the bone marrow. In tumors, IL34 can perform pleiotropic functions depending on the cellular and molecular context.^8^^,^^9^ Four different receptors for this cytokine have been described (CSF1R, TREM2, Syndecan-1, and PTP-ζ), and they are likely to activate distinct signaling pathways.^10^^,^^11^ For example, in lung and colon cancers, IL34 sustains tumor growth by promoting cancer cell proliferation and survival through CSF1R activation,^12^^,^^13^ whereas in glioblastoma cells, IL34 inhibits tumor proliferation through PTP-ζ activation.^14^ The expression of *IL34* is heterogeneous among cancer types, and high IL34 levels can represent either a poor prognostic factor (i.e., liver and lung cancers)^15^^,^^16^ or a favorable prognostic factor (i.e., cervical or head and neck cancers; see the “prognostic summary” of IL34 at the Human Protein Atlas: www.proteinatlas.org). The function of IL34 in RCC has never been described.

In this work, we undertook different approaches to investigate the role of IL34 in the biology and therapeutic response of metastatic RCC. In both the KIRC-TCGA and the UroCCR cohorts, high *IL34* expression correlated with an increased tumor stage, an increased incidence of distant metastases, and a reduced survival in patients. In a syngeneic mouse model, the IL34-enriched TME attracted more monocyte-derived tumor-associated macrophages (MD-TAMs), which negatively altered tumor immunity and vasculature, the two protumor features targeted by current RCC therapy. Mechanistically, the IL34-CSF1R axis promoted their migration rather than enhancing their proliferation. Furthermore, single-nucleus RNA sequencing (snRNA-seq) analysis of MD-TAM cluster revealed that an IL34-enriched TME changed the expression of genes with potential roles in driving immunosuppression and vascular alterations. Finally, in a mouse model of metastatic RCC, blockade of the IL34-CSF1R axis with the Food and Drug Administration (FDA)-approved drug pexidartinib prevented MD-TAMs accumulation in the TME and improved the response to sunitinib or anti-PD1 therapy.

## Results

### IL34 expression is associated with cancer progression in a mouse model of RCC and in patients

**Figure 1:**
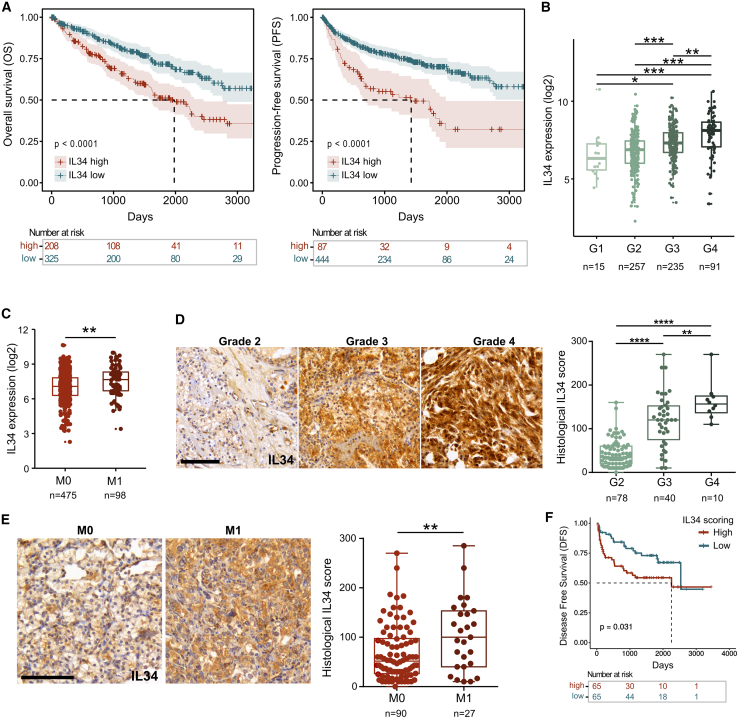
Analysis of IL34 expression in RCC patients (KIRC-TCGA and UroCCR cohorts) (A) Kaplan-Meier curves showing overall and progression-free survival in the KIRC-TCGA patient cohort. An optimal cutoff point was used to split the data into two groups on the basis of high and low IL34 gene expression. Log rank test, *p* values are indicated in the figure. (B and C) (B) Gene expression of IL34 in patient tumors stratified according to Fuhrman grade in the TCGA cohort. Median, min to max. Kruskal-Wallis test. (C) Gene expression of IL34 in patient tumors stratified according to the presence of distant metastases in the TCGA cohort. Median, min to max. Permutation Student’s *t* test. (D) (Left) Representative images of histological analysis of IL34 expression in RCC patient tumors from the UroCCR cohort. (Right) Quantification of the IL34 score stratified according to Fuhrman grade. Median, min to max. One-way ANOVA. Scale bar, 100 μm. (E) (Left) Representative images of IL34 expression in RCC patient tumors (UroCCR cohort). (Right) Quantification of the IL34 score on the basis of the absence or presence of distant metastases (M0 or M1, respectively). Median, min to max. Mann-Whitney U test. Scale bar, 100 μm. (F) Kaplan-Meier curves showing the progression-free survival of patients in the UroCCR cohort. The median value was used to stratify patients into two groups on the basis of high and low histological IL34 scores. Log rank test, *p* values are indicated on the figure. For all panels, number of patients per group and *p* values are indicated on the figure; ∗*p* < 0.05, ∗∗*p* < 0.01, ∗∗∗*p* < 0.001, ∗∗∗∗*p* < 0.0001.

In our previous work,^7^ we identified *IL34* as a potential biomarker of RCC progression, as its expression was gradually upregulated in serially implanted Renca cells in a cell passage-dependent manner (Figure S1A) and was concomitant with an increase in tumor aggressiveness. The expression of the *Csf1* gene, which encodes the other ligand of CSF1R, remained unchanged. Secreted IL34 was increased in the supernatant of Renca cells isolated from the latest passage (P6) compared with that of the parental (P0) cells (Figure S1B) and in the plasma of the serially passaged tumor-bearing mice (Figure S1C). In the KIRC-TCGA cohort, increased *IL34* expression was associated with reduced survival in RCC patients (Figure 1A), increased tumor grade, and increased incidence of distant metastases (Figures 1B and 1C). These clinical findings were further corroborated by histological analysis via tissue microarrays of primary tumors from RCC patients at Bordeaux University Hospital (UroCCR cohort, Table 1). Consistently, high IL34 protein levels were significantly associated with a higher Fuhrman grade, incidence of distant metastases, and reduced patient survival (Figures 1D–1F).

**Table 1:** UroCCR patients

	*n* = 128 (%)	IL34 score	
		Low, *n*	High, *n*
**Sex**			

M	76 (59.4)	40	36
F	41 (32.0)	19	22
N/A	11 (8.6)		

**Age**			

>60	60 (46.9)	31	29
<60	57 (44.5)	28	29
N/A	11 (8.6)		

**TNM**			

T1-T2	77 (60.1)	40	37
T3-T4	40 (31.3)	19	21
N/A	11 (8.6)		
N0	106 (82.8)	58	48
N1-N2	10 (7.8)	1	9
N/A	12 (9.4)		
M0	90 (70.3)	51	39
M1	27 (21.1)	8	19
N/A	11 (8.6)		

**Fuhrman grade**			

G1	0 (0.0)	0	0
G2	78 (60.9)	58	20
G3	40 (31.3)	7	33
G4	10 (7.8)	0	10

F, female; M, male; N/A, not applicable.

### Tumor immunity is the stromal component most impacted by an IL34-enriched TME

**Figure 2:**
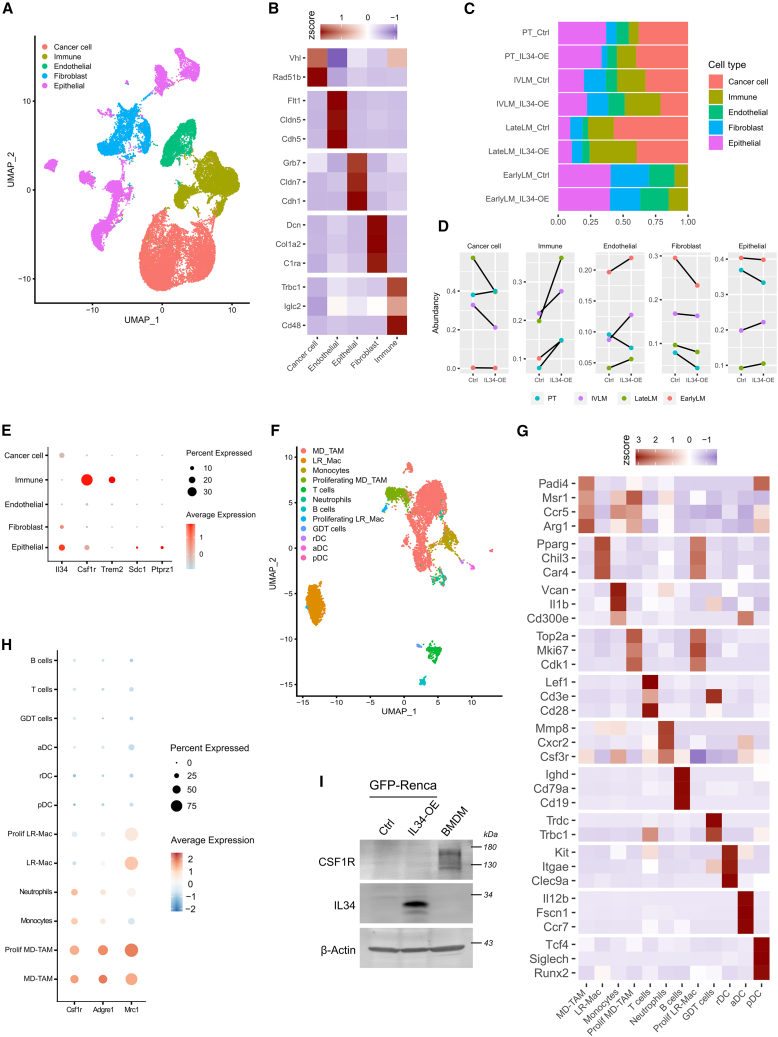
Single-nucleus RNA-seq of primary tumors and lung metastases generated from GFP-Renca cells (A) UMAP representation of the major cell types of 39,681 cells. (B) Heatmap showing the normalized expression of marker genes for each major cell type. (C) Fraction of cell type composition per library. PT, primary tumor; IVLM, intravenous-generated lung metastasis; LateLM, late-stage tumor-derived lung metastasis; EarlyLM, early-stage tumor-derived lung metastasis. (D) Abundancy of each cell type in control (Ctrl) and IL34-overexpressing (IL34-OE) Renca-injected mice. (E) Dot plot showing the expression of Il34, Csfr1, Trem2, Sdc1, and Ptprz1 per cell type. (F and G) (F) UMAP representation of the subclustering of the 7,207 cells in the immune compartment. (G) Heatmap showing the normalized expression of marker genes for each immune subtype. (H) Dot plot showing the expression of Csfr1, Adgre1, and Mrc1 per immune subtype. (I) Western blot analysis of CSF1R protein expression in murine Renca cancer cells and BMDMs.

To gain insight into the protumor mechanisms occurring in an IL34-enriched TME, we generated GFP-Renca cells overexpressing IL34 (IL34-OE) or a control vector (Ctrl, Figure S1D). *In vitro* cell proliferation assays via crystal violet staining revealed that secreted IL34 did not affect the proliferation of Renca cells, ruling out an autocrine mechanism for tumor progression (Figure S1E). Similar results were also obtained using the human RCC cell line 786-O (Figures S1F and S1G). Therefore, we hypothesized that stromal components of the TME might drive IL34-dependent cancer progression. To this end, we performed snRNA-seq analysis of tumors or lung metastases generated via a syngeneic mouse model of RCC to explore the biological impact of an IL34-enriched TME. Either GFP-Renca Ctrl or IL34-OE cells were orthotopically implanted for primary tumor generation (i.e., PT_Ctrl and PT_IL34-OE) or injected into the tail vein to study the TME of lung metastasis (i.e., IVLM_Ctrl and IVLM_IL34-OE). Of the 10 orthotopically implanted mice 2 underwent primary tumor resection, and after 2 additional weeks, tumor-derived lung metastases were collected (i.e., LateLM_Ctrl and LateLM_IL34-OE). The lungs of mice that did not survive tumor resection were also collected to gain insights into the early steps of the lung metastatic process (i.e., EarlyLM_Ctrl and EarlyLM_IL34-OE). In these samples, no visible nodules were observed at the time of sacrifice. Samples from up to 5 mice per group were pooled and processed for nuclear isolation and cDNA library construction (Figure S2). By snRNA-seq analysis, we annotated 5 major clusters: cancer cells, endothelial cells, epithelial cells, fibroblasts, and immune cells (Figures 2A and 2B). Fractions of identified cellular populations differed between samples on the basis of implantation-extraction modality (Figure 2C). On the other hand, only immune cells were consistently affected by the overexpression of IL34 in all modes of implantation (Figure 2D). Among the known receptors of IL34, Csf1r was predominantly expressed in the immune cluster, indicating that the IL34-CSF1R axis could be responsible for the regulation of the tumor immune microenvironment (Figure 2E). Therefore, we further analyzed the immune compartment and identified 12 major clusters: MD-TAMs, lung-resident macrophages (LR-Mac), monocytes, proliferating MD-TAM and LR-Mac, neutrophils, three different populations of dendritic cells (resting, activated, and plasmacytoid), T cells, B cells, and gamma-delta T cells (Figures 2F and 2G). Among immune populations, Csf1r was highly expressed in the myeloid compartment, especially in MD-TAM (Figure 2H). Western blot analysis of tumor cells and murine bone marrow-derived macrophages (BMDMs) confirmed that CSF1R was expressed in monocyte-derived macrophages (Figure 2I). Furthermore, in primary tumors and lung metastases, histological co-staining of CSF1R-positive cells with either F4/80 (an MD-TAM marker) or Ly6G (a neutrophil marker) revealed that the majority of CSF1R-positive cells were MD-TAMs (Figure S3A). Taken together, these data suggest a potential role for the IL34-CSF1R axis in RCC tumor progression through its impact on the biology of monocyte-derived TAMs in the TME.

### An IL34-enriched TME is characterized by the accumulation of MD-TAMs in different preclinical models of RCC

**Figure 3:**
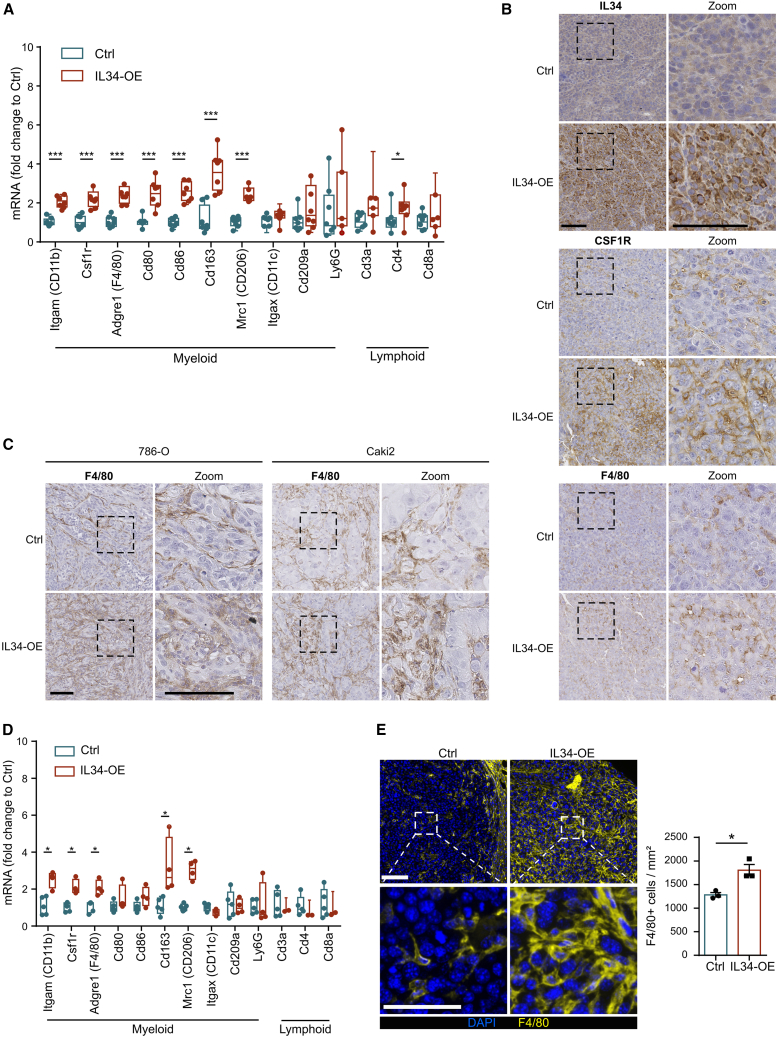
IL34 regulates the immune TME by accumulating monocyte-derived TAMs in primary tumors and lung metastases (A) RT-qPCR analysis of bulk RNA extracted from whole mouse renal tumors generated from IL34-OE (n = 5–7 mice per group) or Ctrl (n = 5–7 mice) Renca cells. Median, min to max. Mann-Whitney U test, ∗*p* < 0.05, ∗∗∗*p* < 0.001. (B) Immunohistochemistry (IHC) showing the expression of IL34, CSF1R, or F480 in Renca-generated orthotopic tumors. Scale bar, 100 μm. (C) IHC images of F4/80-positive cells in orthotopic xenografts generated from the human RCC cell lines 786-O and Caki2. Scale bar, 100 μm. (D) RT-qPCR analysis of bulk RNA extracted from metastatic lungs after tail vein injection of IL34-OE (n = 3–4 mice) or Ctrl (*n* = 5 mice) Renca cells. Median, min to max. Mann-Whitney U test, ∗*p* < 0.05. (E) IHC (left) and quantification (right) of F4/80-positive cells in lung metastases (*n* = 3 mice per group). Scale bar, 100 μm; zoom, 50 μm. Mean ± SEM. Unpaired Student’s *t* tests, ∗*p* < 0.05.

To further corroborate our snRNA-seq data, we analyzed the expression of specific markers for different cell populations involved in innate and adaptive immunity via RT-qPCR of Renca-generated tumors. Overexpression of *IL34* mainly upregulated the expression of markers of the myeloid lineage (e.g., Itgam/CD11b), particularly MD-TAMs (e.g., Adgre1-F4/80 or Csf1r; Figure 3A). Histological analysis revealed that IL34-OE tumors were enriched in MD-TAMs compared with control tumors (Figure 3B). The levels of CSF1, the other ligand of CSF1R, remained unchanged in IL34-OE cancer cells (Figure S1D), indicating that the increase in MD-TAMs was a direct consequence of an IL34-enriched TME. IL34-dependent accumulation of MD-TAMs was also observed in xenograft models of renal tumors generated from the human RCC cell lines 786-O and Caki2 (Figure 3C). Similarly, analyses of IL34-OE lung metastases also revealed increased expression of markers for the myeloid lineage (Figure 3D) and a greater number of MD-TAMs than controls (Figure 3E).

### The IL34-CSF1R axis regulated MD-TAMs accumulation by enhancing their migration

**Figure 4:**
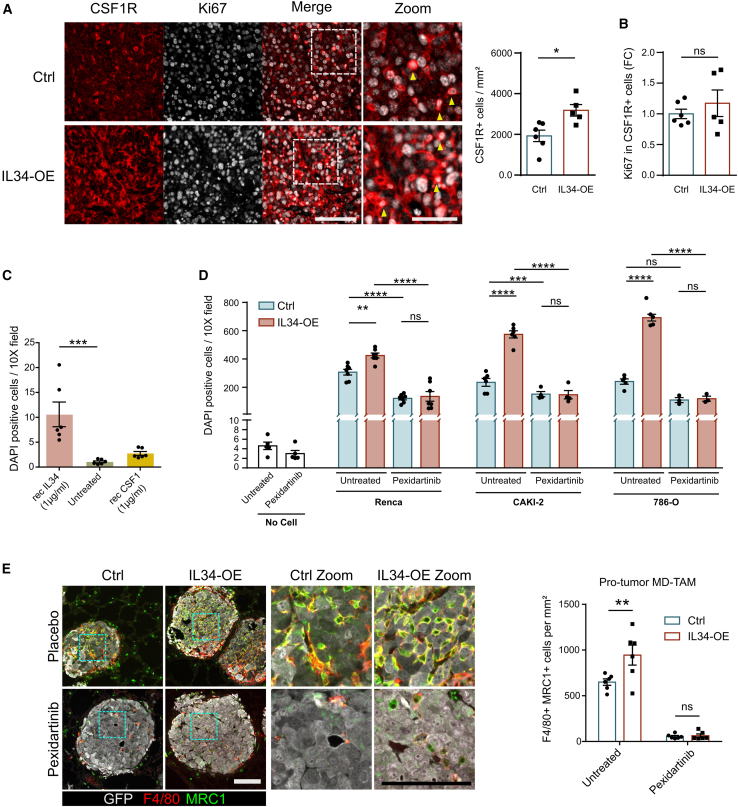
IL34-CSF1R axis is involved in MD-TAMs migration (A) Immunofluorescence analysis of proliferative MD-TAMs (i.e., Ki67 expression in CSF1R+ cells) in primary tumors generated from IL34-OE (*n* = 5 mice) or Ctrl (*n* = 6 mice) Renca cells. The graph on the right shows the quantification of total CSF1R+ cells. Scale bar, 100 μm; zoom, 50 μm. Mean ± SEM. Unpaired Student’s *t* tests, ∗*p* < 0.05. (B) Quantification of proliferative MD-TAMs shown in (A). Mean ± SEM. Unpaired Student’s *t* tests. ns, non significant. FC, fold change. (C) Transwell migration assay of BMDM (*n* = 6 mice per group) treated with either recombinant mouse IL34 or CSF1 (1 μg/mL, 24 hours). One dot represents a single-cell line of BMDM extracted from one mouse. Mean ± SEM. Kruskal-Wallis test, ∗∗∗*p* < 0.001. (D) Transwell migration assay of BMDM after 24 h of coculture with Renca (*n* = 7 mice), 786-O (n = 3–5 mice) or Caki2 (n = 4–6 mice) cells overexpressing mouse IL34 or an empty vector as a control under untreated or pexidartinib-treated (2.5 μM, 24 hours) conditions. One dot represents a single-cell line of BMDM extracted from one mouse. Mean ± SEM. two-way ANOVA, ∗∗*p* < 0.01, ∗∗∗*p* < 0.001, ∗∗∗∗*p* < 0.0001. (E) Histological analysis (left) and quantification (right) of protumor MD-TAMs by counting MRC1+ cells among F4/80+ cells (*n* = 6 mice per group). Scale bar, 100 μm. Mean ± SEM. two-way ANOVA, ∗∗*p* < 0.01.

We investigated whether IL34 could drive MD-TAMs accumulation by increasing their proliferation or monocyte migration in the TME through CSF1R. As expected, histological analysis revealed that the number of CSF1R-positive cells was greater in the IL34-enriched TME than in the control samples (Figure 4A). However, quantification of proliferating CSF1R-positive (Figure 4B) or MRC1-positive cells (known markers for mature MD-TAMs, Figure S3B) by Ki67 staining excluded an effect of IL34 on TAM proliferation. We next investigated the role of IL34 in stimulating MD-TAMs migration. To this end, we performed a transwell migration assay using murine BMDMs. First, by treating BMDMs with 1 μg/mL recombinant mouse IL34 protein, we demonstrated that IL34 could directly increase their migration *in vitro* (Figure 4C). Coculture with either mouse or human RCC cell lines confirmed that IL34-OE cancer cells could enhance BMDMs migration. Conversely, the IL34-driven migration of BMDMs was abolished upon treatment with the CSF1R inhibitor pexidartinib (Figure 4D). Next, to contrast IL34-dependent MD-TAMs accumulation *in vivo*, we undertook a pharmacological approach to inhibit their recruitment to the TME of lung metastases. To this end, the mice were treated with 40 mg/kg pexidartinib for up to 2 weeks and sacrificed the same day. Immunohistochemistry (IHC) analysis confirmed that IL34-enriched metastases had a significantly increased number of MRC1-expressing F4/80+ TAMs (Figure 4E). F4/80+ MRC1+ TAMs are known to have protumor functions and are indicative of MD-TAM (Figure 2H). In pexidartinib-treated mice, the number of protumor MD-TAMs strongly decreased and their IL34-dependent accumulation was not observed. Importantly, in tumor-free mice, pexidartinib treatment was safe at concentrations up to 40 mg/kg since it did not affect the body weight (Figure S4A). Next, using a blood analyzer, we counted the number of circulating leukocytes to estimate the potential effects of systemic CSF1R blockade on the hematopoietic system. This analysis revealed that eosinophils and granulocytes were not significantly affected by pexidartinib, whereas monocytes were reduced by up to 50% in treated mice (Figure S4B). In addition, fluorescence-activated cell sorting analysis of circulating monocytes revealed that pexidartinib did not affect the percentage of vessels patrolling monocytes (i.e., Ly6C^low^) or monocytes able to extravasate into tissues and differentiate into macrophages and DCs (i.e., Ly6C^high^; Figure S4C).^17^ Taken together, these data suggest that depletion of MD-TAMs in lung metastases could be due only in part to systemic myelosuppression and strongly indicate that the IL34-CSF1R axis regulates MD-TAMs accumulation in the TME by enhancing their migration.

### The IL34-enriched TME contributed to tumor immunosuppression and vascular leakage through MD-TAMs accumulation

**Figure 5:**
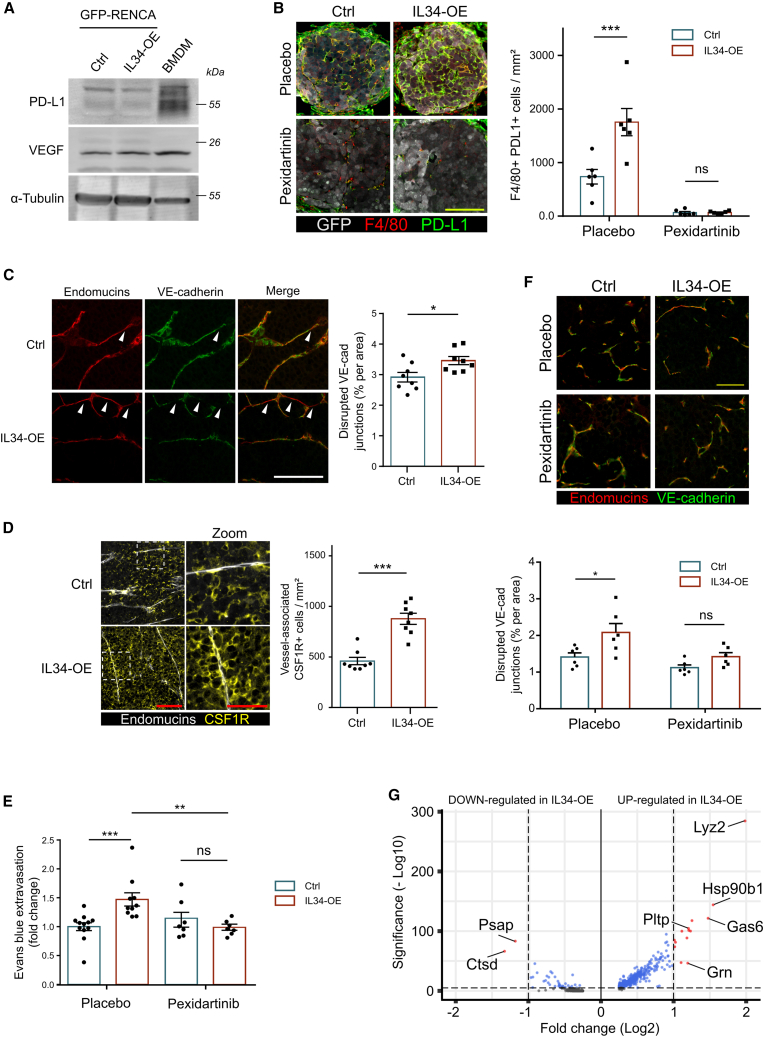
Impact of the IL34-CSF1R axis in the immune-vascular crosstalk of RCC (A) Western blot analysis showing the levels of PD-L1 and VEGF expression in GFP-Renca cells and BMDM. (B) Histological analysis (left) and quantification (right) of immunosuppressive PD-L1+ F4/80+ MD-TAMs in lung metastases (*n* = 6 mice per group). Scale bar, 100 μm. Mean ± SEM. two-way ANOVA, ∗∗∗*p* < 0.001. (C) Primary tumor analysis revealed leaky vessels that were positive for endomucin and expressed low VE-cadherin, as indicated by white arrows (*n* = 8 mice per group). Scale bar, 100 μm. Mean ± SEM. Mann-Whitney U test, ∗*p* < 0.05. (D) Histological images of CSF1R+ cells associated with vessels (endomucin staining) in Renca-generated primary tumors (left) and quantification (right). Scale bar, 100 μm; zoom, 50 μm. Mean ± SEM (*n* = 8 mice per group). Mann-Whitney U test, ∗∗∗*p* < 0.001. (E) Quantification of extravasated Evans blue in Renca-generated primary tumors treated with pexidartinib (40 mg/kg) or placebo. Up to 12 mice per group from two independent experiments were analyzed. Data are expressed as the fold change compared with placebo control samples with tumor weight normalization. Mean ± SEM. two-way ANOVA, ∗∗*p* < 0.01, ∗∗∗*p* < 0.001. (F) Histological images of leaky vessels (top) and quantification (bottom) of lung metastases from mice treated with pexidartinib (40 mg/kg) or placebo (*n* = 6 per group). Scale bar, 50 μm. Mean ± SEM. two-way ANOVA, ∗*p* < 0.05. (G) Volcano plot of the DEGs identified via MD-TAM. The x axis represents the log2-fold change in gene expression between the IL34-OE and Ctrl groups, indicating upregulation (right) and downregulation (left) in IL34-OE MD-TAM. The y axis represents the −log10 of the adjusted *p* value. The horizontal dashed line indicates the threshold for statistical significance (i.e., adjusted *p* value <0.05).

TAMs are known to promote cancer progression and resistance to therapy by expressing molecules that can alter tumor immunity and vasculature, the two major targets of RCC therapy. We thus tested whether the IL34-driven accumulation of TAMs could negatively impact these two stromal components. First, murine BMDMs expressed PD-L1 and VEGF, two main protumor proteins that mediate immunosuppression, tumor angiogenesis, and vessel permeability, respectively (Figure 5A). In accordance with our data, in IL34-enriched lung metastases, the number of F4/80+PD-L1+ TAMs was significantly greater than that in controls, whereas pexidartinib treatment strongly reduced their recruitment and abolished the IL34-dependent accumulation observed in untreated mice (Figure 5B). Next, we analyzed the tumor vasculature in our murine models. A typical VEGF-related feature of a reduced response to therapy is decreased tumor vasculature functionality or increased vessel permeability.^18^^,^^19^ Histological analysis of the vasculature of primary tumors revealed a reduction in VE-cadherin expression in IL34-OE samples, a sign of augmented vascular leakage (Figure 5C). Importantly, this phenotype was associated with an increase in vessel-associated CSF1R-positive cells (Figure 5D), indicative of enhanced recruitment of MD-TAMs. Next, we corroborated these results by performing an *in vivo* Miles assay to assess the role of IL34-CSF1R axis in regulating tumor vascular permeability. Compared with control tumors, vessel leakage was significantly increased in IL34-OE samples. In pexidartinib-treated mice, no IL34-dependent increase in vessel permeability was observed (Figure 5E), suggesting that IL34-driven vascular dysfunction in untreated mice was generated by increased recruitment of MD-TAMs. Also, in the placebo group, the growth of IL34-OE primary tumors was augmented than control mice, whereas we did not observe such a difference in treated mice (Figure S5A). Next, IHC analysis of vascular endothelial cadherin (VE-cadherin) expression in vessels of lung metastases confirmed the data obtained in primary tumors (Figure 5F). Taken together, our results confirmed the involvement of the IL34-CSF1R axis in RCC progression through the regulation of the immune-vascular crosstalk in the TME. Finally, we investigated whether an IL34-enriched TME could polarize MD-TAMs into a protumor phenotype. Analysis of the differentially expressed genes (DEGs) in the MD-TAM cluster revealed that an IL34-enriched TME regulated the expression of various genes with either known or unknown roles in TAM or cancer progression (Tables S1 and S2). In particular, within the IL34-OE samples, genes whose expression was upregulated more than 2-fold included *Lyz2*, *Hsp90b1*, *Gas6*, *Pltp,* and *Grn*. Conversely, only the *Psap* and *Ctsd* genes were downregulated more than 2-fold (Figure 5G). These genes are known to have different cellular functions, including the regulation of pro- or antitumor phenotypes in TAMs.^20^^,^^21^^,^^22^^,^^23^^,^^24^^,^^25^^,^^26^^,^^27^^,^^28^

### Pexidartinib, in combination with sunitinib or anti-PD1 therapy, improved therapeutic outcome in the metastatic RCC model

**Figure 6:**
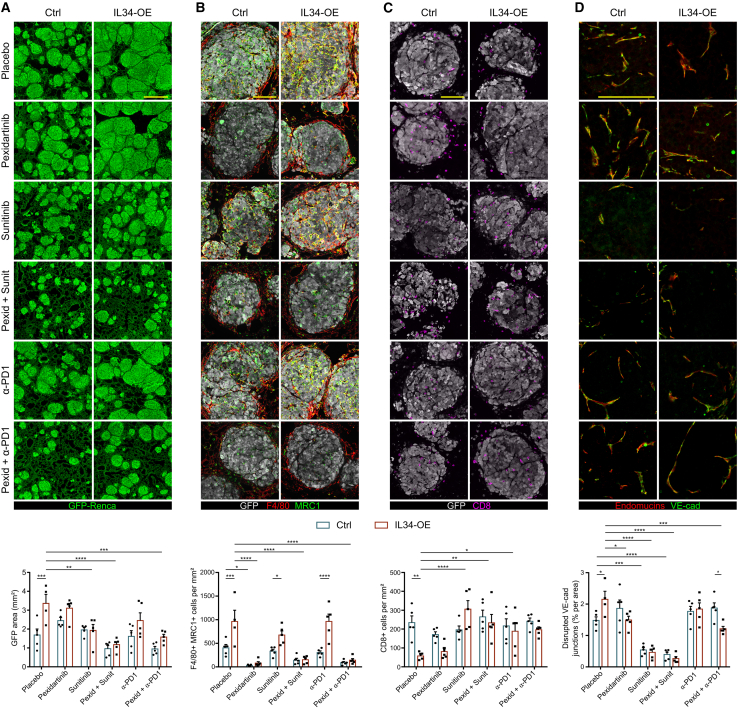
Metastatic growth in mice treated with pexidartinib alone or with standard RCC therapy (A–D) Histological images (top) and quantification (bottom) of the GFP area (A; scale bar. 500 μm), protumor MD-TAMs recruitment (B; scale bar, 100 μm); infiltrating cytotoxic CD8^+^ T cells (C; scale bar, 100 μm), and leaky vasculature (D; scale bar, 100 μm) in the metastatic lungs of mice treated with pexidartinib either alone or in combination with sunitinib or an anti-PD1 antibody; 4–5 mice per group were analyzed. Mean ± SEM. two-way ANOVA, ∗*p* < 0.05, ∗∗*p* < 0.01, ∗∗∗*p* < 0.001, ∗∗∗∗*p* < 0.0001.

We then tested pexidartinib as a cotreatment for metastatic RCC in our preclinical model. To this end, 4 days after the tail vein injection of Ctrl or IL34-OE Renca cells, we treated the mice for 2 weeks with pexidartinib alone or in combination with sunitinib or anti-PD1 (i.e., the 2 first-line drugs for metastatic RCC) and measured the size of the metastases in the lungs via GFP staining (Figure 6A). In the placebo group, the surface covered by IL34-OE metastases was significantly 2 times larger than that in the control group (mean GFP area: 1.68 vs. 3.36 mm^2^), confirming the protumor role of IL34. This difference was eliminated in all the treated mice. Compared with placebo, pexidartinib or anti-PD1 monotherapy did not significantly reduce metastatic growth, whereas sunitinib alone was effective only in the IL34-OE groups (3.36 vs. 1.93 mm^2^). However, compared with the placebo, the combination of pexidartinib with either sunitinib or an anti-PD1 antibody significantly reduced metastatic growth in IL34-OE-treated mice. In particular, in Ctrl mice, the mean GFP-positive area slightly decreased between placebo and combined therapy (1.68 vs. 0.96 or 0.95 mm^2^ for placebo vs. Pexid-Sunit or Pexid-aPD1, respectively), whereas in IL34-OE mice, the GFP area was almost 3 times reduced in Pexid-Sunit mice (3.36 vs. 1.18 mm^2^) and 2.1 times reduced in the Pexid-aPD1 group (3.36 vs. 1.58 mm^2^). To gain insight into the TME changes that could mediate the therapeutic effects, we subsequently analyzed the infiltration of protumor MD-TAMs and the levels of immunosuppression and vascular dysfunction. First, we observed a significant reduction in the number of protumor MD-TAMs in pexidartinib-treated mice, either alone or in combination with sunitinib or anti-PD1 therapy (Figure 6B). The infiltration of cytotoxic CD8^+^ T cells was unchanged among control mice and in IL34-OE mice treated with pexidartinib alone. However, sunitinib or anti-PD1 therapy, either alone or in combination with pexidartinib, significantly increased the number of T cells in the IL34-OE metastases (Figure 6C). Finally, compared with placebo, sunitinib treatment normalized the metastatic vasculature either alone or in combination with pexidartinib, whereas in IL34-OE mice, such an effect was also observed after pexidartinib treatment (Figure 6D).

### In RCC patients, increased IL34 expression is correlated with MD-TAMs infiltration, immunosuppression, and resistance to anti-PD1 therapy

**Figure 7:**
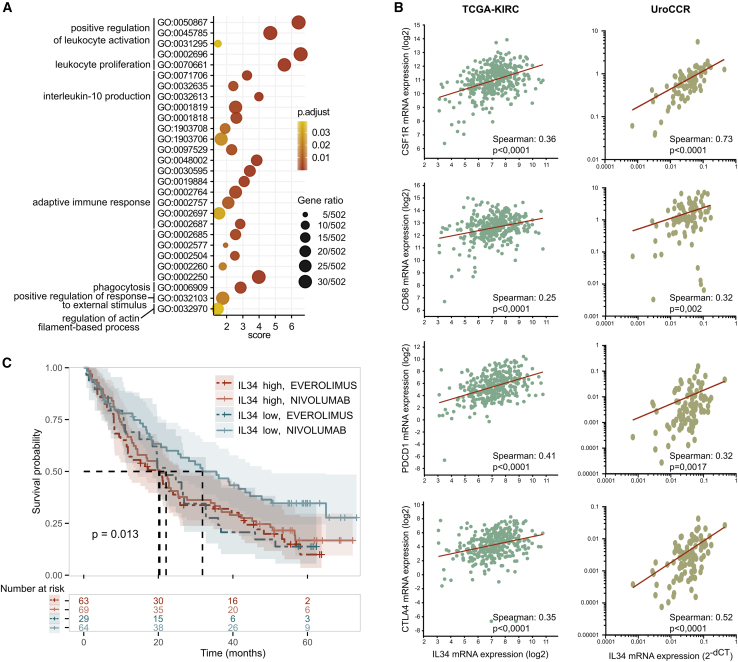
In RCC patients, high *IL34* expression correlates with features of immunosuppression and a reduced response to immunotherapy (A) Gene Ontology term enrichment analysis of genes whose expression correlated with IL34 expression in KIRC-TCGA patients. (B) Spearman correlation analysis of *IL34* expression with the MD-TAM markers *CSF1R* and *CD68* or the exhausted T cell markers *PDCD1* and *CTLA4* in the KIRC-TCGA (left) and UroCCR (right) databases. (C) Kaplan-Meier survival analysis of patients stratified on the basis of *IL34* expression (high vs. low) who received treatment (everolimus vs. nivolumab, CheckMate CM-025 cohort). Number of patients per group and *p* values are indicated in the figure.

To test the translational significance of our findings, we analyzed various patient cohort databases. By performing Gene Ontology (GO) enrichment analysis, we determined that the genes whose expression significantly correlated with *IL34* expression in the KIRC-TCGA database were involved in immune-related biological processes (Figure 7A). This analysis confirmed that IL34 has a major effect on the regulation of the tumor immune microenvironment in RCC patients. In particular, in line with our findings, *IL34* expression correlated with the expression of *CSF1R* or *CD68* (markers for myeloid cells and human macrophages, respectively) in both the KIRC-TCGA and UroCCR cohorts (Figure 7B). We also analyzed the correlation between *IL34* and *CD274* (coding for PD-L1) expressions and found a positive correlation in the UroCCR cohort. However, in the TCGA cohort this correlation was inverted (Figure S6A), indicating that in real world *CD274* gene status might not be the ideal marker to identify immunosuppressive TAM in human RCC. Instead, we found that *IL34* expression positively correlated with a gene signature specific to immunosuppressive TAM of RCC patients (i.e., CD38+MSR1+MRC1-, Figure S6B).^29^ Furthermore, IL34 expression correlated with *PDCD1* or *CTLA4* (markers of T cell exhaustion) in both the KIRC-TCGA and the UroCCR cohorts (Figure 7B). Finally, we investigated whether high levels of IL34 in patients can affect the response to nivolumab, an anti-PD1 antibody used to treat metastatic RCC. To this end, we analyzed the CheckMate CM-025 cohort by stratifying patients based on the expression of IL34 in the two treated groups (i.e., nivolumab or everolimus).^30^ Patients with high *IL34* expression were less responsive to anti-PD1 therapy than patients with low *IL34* expression (Figure 7C). The expression of *IL34* was not predictive of the response to the previous RCC standard-of-care mTOR inhibitor everolimus, which exerts a wide range of anti-neoplastic effects by targeting cancer and stromal cells.^31^ Notably, the levels of the twin cytokine *CSF1* were not predictive of therapy response (Figure S6C). Thus, these data highlight the importance of IL34 in mediating levels of immunosuppression and, in particular, response to immunotherapy in RCC patients.

## Discussion

Although surgical resection offers a viable option for patients with localized RCC tumors, patients with metastatic RCC (i.e., one-third of diagnosed cases) still lack effective alternatives. The failure of treatment resides in the absence of validated biomarkers and limited knowledge of the biological processes occurring during RCC progression.^4^^,^^6^^,^^32^ Building on our previous work, which identified IL34 as a potential biomarker, we have investigated the role of IL34 in RCC biology to address an unmet medical need. In this work we have provided a comprehensive analysis of the IL34-CSF1R axis in the regulation of the immune-vascular crosstalk both in the primary tumors and metastases of RCC.

In RCC patients, *IL34* expression is increased in late-stage tumors and is correlated with worse survival. In contrast to previous studies, our experiments excluded IL34-dependent effects on cancer cell proliferation, which could explain patient outcomes. In fact, the IL34-CSF1R axis was demonstrated to sustain tumor cell growth and survival via an autocrine effect in different preclinical tumor models.^12^^,^^13^^,^^33^ Here, using a validated mouse model for preclinical studies of RCC, the upregulation of *IL34* in cancer cells led to the accumulation of monocyte-derived TAMs through CSF1R. Interestingly, the IL34-CSF1R-dependent accumulation of MD-TAMs originated from enhanced chemotaxis of circulating monocytes rather than a proliferative effect on TAMs. The role of the IL34-CSF1R axis in macrophage migration was also observed during embryogenesis in two different studies conducted in zebrafish.^34^^,^^35^ In cancer, using a model of osteosarcoma, Ségaliny et al. reported that IL34 promoted TAM recruitment into tumors, possibly by enhancing monocyte adhesion to activated human umbilical vein endothelial cells (HUVEC) monolayers via a CSF1R-independent mechanism.^36^ In the literature, evidence of the direct involvement of the IL34-CSF1R axis in TAM migration, especially in the TME of the metastasis, is unclear. Different studies showed a positive correlation between IL34 and pro-tumor TAMs infiltration in primary tumors. However, this was mainly due to the role of IL34 in altering TAMs polarization toward the pro-tumor M2-type phenotype.^12^^,^^37^^,^^38^^,^^39^^,^^40^

TAMs are generally categorized into two main functionally contrasting subtypes: the proinflammatory M1 type, which typically performs antitumor functions, and the anti-inflammatory M2 type, which leads to tumor progression by promoting tumor occurrence and metastasis and tumor angiogenesis and inhibiting the T-cell-mediated antitumor immune response.^41^ Previous studies have demonstrated the role of IL34 in regulating TAMs polarization toward a protumor phenotype through the upregulation of classical M2-type markers (e.g., MRC1/CD206).^42^ Our snRNA-seq analysis revealed a potential new gene signature of protumor MD-TAM polarization driven by IL34. For example, the genes whose expressions were most upregulated were *Lyz2*, *Hsp90b1*, *Gas6*, *Pltp,* and *Grn*. Although the functions of *Gas6*, *Pltp,* and *Grn* in regulating the angiogenic and immunosuppressive properties of TAMs have been previously described (see Table S2), the roles of *Lyz2* and *Hsp90b1* in these cells remain elusive. *Lyz2* is highly expressed in monocyte-derived macrophages and promotes neuronal repair after optic nerve injury.^20^ This tissue repair behavior aligns with the classical characteristics of anti-inflammatory or M2-type TAMs. *Hsp90b1* expression is correlated with poor prognosis, advanced stages, and immunosuppression in different types of cancer, including RCC.^21^ However, *Hsp90b1* expression and function in TAMs have never been described. On the other hand, an IL34-enriched TME downregulated *Psap* and *Ctsd* genes, whose expression is associated with proinflammatory pathways,^27^^,^^28^ which are often activated in M1-type TAMs. This analysis revealed that the IL34-CSF1R axis could also perform protumor functions by regulating MD-TAMs behavior in addition to their recruitment to the TME. Further studies are needed to address whether IL34 can drive such polarization by directly interacting with the MD-TAMs population or through another stromal component.

The increase in MD-TAMs infiltration in solid tumors correlates with resistance to therapy in most cancers through various mechanisms.^43^ In this work, MD-TAMs contributed to increased immunosuppression and vessel permeability, two primary protumor features that hinder cancer therapy. In RCC patients, *IL34* expression was correlated with the expression of markers of TAMs and exhausted T cells in two independent patient cohorts (UroCCR and KIRC-TCGA). In addition, analysis of the CheckMate 025 cohort indicated that, unlike *CSF1*, *IL34* expression could predict the response to anti-PD1 therapy. These analyses underscore the translational relevance of our findings and indicate a potential role for IL34 in the interplay between immunosuppressive TAMs and cytotoxic T cells in RCC patients. In line with these findings, Hama et al. reported an immunosuppressive protumor role for IL34 in a murine model of melanoma.^40^ Here, the IL34-enriched TME was characterized by the accumulation of immunosuppressive MD-TAMs, a reduction in cytotoxic CD8^+^ T cell infiltration, and increased vessel permeability in mouse renal tumors and metastases. In addition, augmented vascular leakage was associated with an increase in vessel-associated CSF1R-positive cells, which are likely to represent perivascular MD-TAMs. In line with the known protumor activities of perivascular TAMs in facilitating angiogenesis and vascular permeability,^44^^,^^45^ we demonstrated that inhibiting the IL34-CSF1R axis with pexidartinib impeded the augmented vascular leakage observed in untreated tumors with an IL34-enriched TME. The role of IL34 in controlling tumor vascular architecture through immune cells was also reported by Kajihara et al. in a mouse model of breast cancer. In particular, they showed that IL34 could alter the response to chemotherapy by negatively regulating the proangiogenic activity of polymorphonuclear myeloid-derived suppressor cells (PMN-MDSC) in the TME of 4T1 subcutaneous tumors.^46^ These findings seemed to be independent of CSF1R, suggesting the involvement of another IL34 receptor. Two main reasons may explain the difference in their findings compared with our results. First, due to the pleiotropic roles of IL34, which depend on the cellular and molecular context of the TME, it is not surprising that IL34 can have different functions in different types of tumor. Second, they used subcutaneous tumors whose TME usually differs from the one of orthotopic tumors.^47^^,^^48^ Taken together, our results reveal the involvement of the IL34-CSF1R axis in the RCC tumor vasculature and immunity through the regulation of MD-TAMs biology and suggest that blockade of the IL34-CSF1R axis could be used to improve RCC therapy.

Although several studies reported the role of IL34 in cancer, we are still missing therapeutic strategies that target IL34-driven mechanisms to implement in the clinics. For this reason, the improved therapeutic effects observed with pexidartinib are interesting from a practical perspective. Since this CSF1R inhibitor is FDA approved,^49^ it can be already tested for the treatment of the metastatic forms of RCC in combination with standard-of-care therapies. Although pexidartinib alone impeded the accumulation of immunosuppressive MD-TAMs in lung metastases, in IL34-OE pexidartinib-treated mice we observed a nonsignificant decrease in infiltrating CD8^+^ T cells, similar to those in the Ctrl group. This finding indicated that other mechanisms of immunosuppression remained in place, explaining the inability of pexidartinib, as a single agent, to generate a more immunostimulant TME. For example, Voissière et al. reported that in advanced cancers, pexidartinib affects the differentiation of antitumor dendritic cells, potentially limiting the effects of anti-PD-L1 therapy.^50^ Additionally, Kumar et al. demonstrated that CSF1R blockade can enhance tumor immunosuppression by recruiting MDSC.^51^ On the other side, we showed that the combination of pexidartinib with sunitinib or an anti-PD1 antibody could exert a synergistic effect in reducing metastatic growth of RCC. Although both types of combination significantly reduced the levels of immunosuppression in the metastatic TME, we observed a reduction in tumor vasculature leakage only in mice treated with pexidartinib and sunitinib. In addition, IL34-OE mice were more responsive to pexidartinib plus sunitinib than their placebo counterparts, highlighting the role of the IL34-CSF1R axis in the immune-vascular crosstalk mediated by increased MD-TAMs recruitment. Taken together, our findings show the importance of evaluating the clinical benefit of pexidartinib in RCC in combination with current therapies.

Several clinical trials tested the beneficial use of CSF1R inhibitors in combination with immunotherapy, targeted therapy, or chemotherapy in different types of cancer. However, these studies led to controversial findings.^52^ Intra- and inter-tumor heterogeneity or the lack of biomarkers that could predict the rate of response to CSF1R inhibitors are potential reasons for such discrepancies in the outcomes observed in clinical trials. The use of pexidartinib in RCC was only tested in a phase Ib study,^53^ but we are still lacking evidence of its potential in treating this type of cancer. Our data suggest the implementation of pexidartinib with current RCC therapy, especially for those patients who express high levels of IL34.

### Limitations of the study

Renca cells do not reflect the pathological features observed in human RCC. In fact, the majority of RCC patients present inactive mutations or deletions of VHL, whereas Renca cells have intact expression of this gene.^2^^,^^54^ However, the Renca model permitted the investigation of the protumor mechanisms of immunosuppression and vessel permeability using an intact TME. Furthermore, similar to tumors of RCC patients, TAMs represented the most abundant myeloid cell population of the TME in our mouse models, allowing us to investigate the role of IL34 in the regulation of this cell population. The effect of IL34 on MD-TAMs recruitment was also validated in the human cell lines 786-O and Caki2 (*VHL*-mutated and VHL-wild-type, respectively). Notably, in the last decade, the Renca syngeneic mouse model has been widely used as the main preclinical model for RCC research, generating a wealth of data that eventually contributed to significantly improving RCC therapy.^55^^,^^56^^,^^57^^,^^58^^,^^59^

Another limitation is the use of a CSF1R inhibitor to block IL34 protumor activities. Using an anti-IL34 antibody could have been a better strategy for ruling out potential interference of CSF1. However, our work would have lost its potential clinical impact: whereas anti-IL34 antibodies have never been tested in humans, pexidartinib is FDA approved and already available to test in patients. Also, in our experimental settings, pexidartinib was likely to block the IL34-driven alterations of the immune-vascular crosstalk, as CSF1 levels were unchanged in IL34-OE cancer cells and not associated with aggressiveness and progression of Renca tumors and metastases. Finally, IL34 showed a greater ability than CSF1 to induce BMDM migration *in vitro*.

## Resource availability

### Lead contact

Further information and any requests should be directed to and will be fulfilled by the lead contact, Prof. Andreas Bikfalvi (andreas.bikfalvi@u-bordeaux.fr).

### Materials availability

This study did not generate new unique reagents.

### Data and code availability

• Gene expression and survival data from patients in the TCGA-KIRC database were acquired from xenabrowser.net and cbioportal.org. The raw sequencing reads from the snRNA-seq analysis have been deposited in EMBL’s European Bioinformatics Institute database (accession number: E-MTAB-14432).
• No new or custom code has been used to analyze the data.
• Additional information (e.g., experimental details) can be obtained from the lead contact upon request.

## Acknowledgments

This work was supported by Siric-BRIO (postdoctoral fellowship to A.E.), Fondation ARC 2019 (ref. PJA 20191209471), Plan Cancer INSERM 2017 (ref. C18005GS), and Conseil Regional d’Aquitaine 2020 and by recurrent funding from 10.13039/501100001677Inserm and the 10.13039/501100006251University of Bordeaux to A.B. The authors thank Xavier Gauthereau (TBMCore, Bordeaux) and Frédéric Martins (GeT-Santé, Toulouse) for their help with snRNA-seq library preparation and sequencing. Resources and services used for snRNA-seq analysis were provided by the VSC (Flemish Supercomputer Center), funded by the 10.13039/501100003130Research Foundation Flanders (FWO) and the 10.13039/501100011878Flemish Government.

## Author contributions

A.B. conceived the study. A.E. designed the experiments. A.E., W.S., Y.P., M.-A.D., J.M., C.M.S., and T.M. performed experiments. A.E., W.S., T.C., B.B., L.C., D.A., and D.L. were involved in data analysis. J.-C.B. collected clinical samples. A.E. and A.B. wrote the manuscript, and the final version of the manuscript was reviewed and approved by all authors.

## Declaration of interests

The authors declare that they have no conflicts of interest.

## STAR★Methods

### Key resources table

**Table:** 

REAGENT or RESOURCE	SOURCE	IDENTIFIER
**Antibodies**		

Goat anti-mouse CSF1	bio-techne	AF416; RRID:AB_355351
Rat anti-F4/80	Invitrogen	14-4801-85; RRID:AB_467559
Mouse anti-F4/80	Santa Cruz	sc-377009; RRID:AB_2927461
Goat anti-MRC1/CD206	bio-techne	AF2535; RRID:AB_2063012
Rabbit anti-GFP	MBL	598; RRID:AB_591816
Rabbit anti-CSF1R	Cell Signaling	#3152; RRID:AB_2085233
Rabbit anti-mouse IL34	antibodies-online	ABIN1175348; RRID: AB_3695706
Mouse anti-human IL34	Abcam	ab101443; RRID:AB_10711208
Rat anti-Ly6G	BD Pharmigen	551459; RRID:AB_394206
Rat anti-Ki67	Invitrogen	14-5698-82; RRID:AB_10854564
Goat anti-Endomucins	bio-techne	AF4666; RRID:AB_2100035
Rat anti-VEcadherin	BD Bioscience	555289; RRID:AB_395707
Mouse anti-PD-L1/CD274	Proteintech	66248-1-Ig; RRID:AB_2756526
Rabbit anti-CD8	Abcam	ab217344; RRID:AB_2890649
Rabbit anti-PD-L1/CD274	RD system	MAB90781; RRID:AB_2921258
Sheep anti-mouse IL34	bio-techne	AF5195; RRID:AB_2124393
Mouse anti-α-Tubulin	Sigma-Aldrich	T6199; RRID:AB_477583
Mouse anti-β-Actin	Santa Cruz	sc-69879; RRID:AB_1119529
Mouse anti-VEGF	Invitrogen	MA5-13182; RRID:AB_10981661
Donkey anti-rabbit Alexa Fluor 488	Jackson ImmunoResearch	711-545-152; RRID:AB_2313584
Donkey anti-rabbit Alexa Fluor 647	Jackson ImmunoResearch	711-605-152; RRID:AB_2492288
Donkey anti-rat TRITC	Jackson ImmunoResearch	712-025-153; RRID:AB_2340636
Donkey anti-rat Alexa Fluor 647	Jackson ImmunoResearch	712-605-153; RRID:AB_2340694
Donkey anti-goat TRITC	Jackson ImmunoResearch	705-025-147; RRID:AB_2340389
Donkey anti-goat Alexa Fluor 647	Jackson ImmunoResearch	705-605-147; RRID:AB_2340437
Donkey anti-mouse Alexa Fluor 647	Jackson ImmunoResearch	715-605-151; RRID:AB_2340863
PE anti-CD45, clone 30-F11	BD Bioscience	553081; RRID:AB_394611
Alexa Fluor 647 anti-SiglecF, clone E50-2440	BD Bioscience	562680; RRID:AB_2687570
PE-Cy7 anti-FceR1, clone MAR-1	Invitrogen	25-5898-82; RRID:AB_2573493
PerCP-eFlour710 anti-CD172a, clone P84	eBiosciences	46-1721-80; RRID:AB_10805866
BV785 anti-CD11b, clone M1/70	BioLegend	101243; RRID:AB_2561373
Pacific Blue anti-Ly6G, clone 1A8	BioLegend	127611; RRID:AB_1877212
APC-Cy7anti-Ly6C, clone HK1.4	BioLegend	128025; RRID:AB_10643867
BV605 anti-CD115, clone AFS98	BioLegend	135517; RRID:AB_2562760

**Bacterial and virus strains**		

Lentivirus pRRLsin-MND-eGFP-WPRE	This paper	N/A
Lentivirus pCMV-puro-IL34	This paper	N/A

**Biological samples**		

UroCCR samples	University Hospital, Bordeaux	NCT03293563

**Chemicals, peptides, and recombinant proteins**		

Pexidartinib	MedChemExpress	HY-16749/CS-4256
Sunitinib	MedChemExpress	HY-10255A/CS-1670

**Critical commercial assays**		

Chromium Next GEM Single Cell 3′ GEM	10X Genomics	Library & Gel Bead Kit v3.1
Mouse IL-34 Quantikine ELISA Kit	RD System	M3400

**Deposited data**		

snRNA-seq	EMBL’s European Bioinformatics Institute database	E-MTAB-14432; RRID:SCR_004727

**Experimental models: Cell lines**		

786-O	ATCC	ATCC CRL-1932; RRID:CVCL_1051
Caki2	ATCC	ATCC HTB-47; RRID:CVCL_0235
Renca	ATCC	ATCC CRL-2947; RRID:CVCL_2174

**Experimental models: Organisms/strains**		

BALB/c mice	Charles River Laboratories	

**Oligonucleotides**		

Primers for qPCR via SYBR green are listed in Table S4	This paper	N/A
Csf1r TaqMan Assay	ThermoFisher Scientific	Mm01266652_m1
Il34 TaqMan Assay	ThermoFisher Scientific	Mm01243248_m1
Csf1 TaqMan Assay	ThermoFisher Scientific	Mm00432686_m1
Forward primer with attB site for IL34 cDNA amplification 5′-GGGG-ACA-AGT-TTG-TACAAA- AAA-GCA-GGC-TTC-ATG-CCC-TGG-GGA-CTC-GCC-TGG-CTA-3′	This paper	N/A
Reverse primer with attB site for IL34 cDNA amplification 5′-GGGG-AC-CAC-TTT-GTA-CAA-GAA-AGC-TGG-GTC-TCA-GGG-CAA-CGA-GCCATG-GCT-TGA-3′	This paper	N/A

**Recombinant DNA**		

pLenti CMV/TO Puro DEST	Addgene	#17293; RRID:Addgene_17293
pVSV-G	Addgene	#8454; RRID:Addgene_8454
psPAX2	Addgene	#12260; RRID:Addgene_12260

**Software and algorithms**		

RStudio		RRID:SCR_000432
GraphPad Prism		RRID:SCR_002798

### Experimental model and study participant details

#### Cell culture

The human cell lines 786-O and Caki2 were purchased from ATCC and cultured in RPMI 1640. Mouse renal carcinoma Renca cells were purchased from ATCC and cultured in DMEM. The media were supplemented with 10% (v/v) fetal bovine serum (Gibco; Thermo Fisher Scientific) and 1% (v/v) penicillin–streptomycin (Sigma‒Aldrich), and the cells were cultured at 37°C in a humidified incubator containing 5% CO2.

#### Animal models

Female BALB/c mice of 8 to 12 weeks (Charles River Laboratories) were housed in the animal facility of Bordeaux University (Animalerie Mutualisée, Université de Bordeaux, France). All studies involving animals were approved by the Ministere de l’Enseignement Supérieur, de la Recherche et de l’Innovation (authorization number #31294-2021042114529886 v3).

#### Human participants

##### UroCCR cohort

Patient samples from the UroCCR cohort were used with associated clinical data (clinicaltrial.gov, NCT03293563). Patients’ information are summarized in Table 1. All patients gave written informed consent. The UroCCR database (French Research Network for Kidney Cancer) is IRB-approved (Comité Consultatif sur le Traitement de l'Information en Matière de Recherche dans le domaine de la Santé) and obtained the CNIL (Commission Nationale de l'Informatique et des Libertés) authorization number DR-2013-206.

### Method details

#### Generation of stable cell lines

Renal cancer cell lines were infected with a lentiviral vector (pRRLsin-MND-eGFP-WPRE, obtained from the vectorology platform of the University of Bordeaux Vect’UB) expressing the eGFP for imaging purposes. GFP-positive cells were then sorted with a FACSAria sorter (BD Biosciences). The mouse IL34 lentiviral vector was obtained by extracting total RNA from Renca cells and reverse-transcribing it into cDNA via a high-capacity cDNA reverse transcription kit (Applied Biosystems, 4368814). Mouse IL34 cDNA was then amplified via PCR via specific primers containing the attB site: fwd: 5′-GGGG-ACA-AGT-TTG-TACAAA- AAA-GCA-GGC-TTC-ATG-CCC-TGG-GGA-CTC-GCC-TGG-CTA-3’; rev: 5′-GGGG-AC-CAC-TTT-GTA-CAA-GAA-AGC-TGG-GTC-TCA-GGG-CAA-CGA-GCCATG-GCT-TGA-3’. The attB-PCR product was subsequently cloned and inserted into pDONR221 (Thermo Fisher, #12536017) via BP gateway cloning and subsequently transferred into pLenti CMV/TO Puro DEST (Addgene #17293) via LR gateway cloning to obtain the pLenti CMV/TO-mIL34-Puro lentiviral vector. Viral particles were produced by calcium phosphate cotransfection of HEK293T cells with the packaging plasmids pVSV-G (Addgene, #8454) and psPAX2 (Addgene, #12260) and a transfer plasmid (e.g., eGFP or mIL34 plasmid). Renca cells were infected with lentiviruses and selected for 7 days in complete DMEM supplemented with 1 μg/mL puromycin. All the constructs were sequence verified.

#### Cell proliferation assay

For the proliferation assay, 2 × 10^5^ Renca or 1 × 10^5^ 786-O cells were seeded in 6-well plates, and the medium was changed every day. At each time point, the cells were gently washed with PBS, fixed with 4% FA for 10 min at RT, and stained with 0.5% crystal violet for an additional 10 min at RT. The cells were then washed several times with tap water and dried completely. Once all the time points were collected, the cells were destained with 1% SDS under agitation. The intensity of the dissolved crystal violet was read at 595 nm via a spectrophotometer.

#### Animal experiments

Syngeneic renal tumors or metastases were established via subcapsular implantation or tail vein injection, respectively. For subcapsular implantation, 1 × 10^5^ Renca cells were injected under the left kidney capsule, whereas for intravenous injection, 5 × 10^5^ Renca cells were injected into the caudal vein. Two to three weeks after implantation/injection, the generated primary tumors or lung metastases were either fixed in 4% formaldehyde for histological analysis or snap frozen in liquid nitrogen for bulk RNA or protein extraction. For *in vivo* treatments, the mice were randomized into different groups and treated four days after Renca cell injection. Pexidartinib or sunitinib (HY-16749/CS-4256 or HY-10255A/CS-1670, MedChemExpress) was dissolved in 5% DMSO - 95% corn oil and administered once daily by gavage (40 mg/kg per mouse) for two cycles, where one cycle consisted of 5 days of treatment. Anti-PD1 antibody (100 μg/mouse, BE0146, BioXCell) was administered by intraperitoneal injection twice per week for a total of two weeks. At least 2 h after the last treatment, the mice were culled for further analysis.

#### Immunohistochemistry and tissue microarrays

Formalin-fixed, paraffin-embedded (FFPE) tissues were cut via a microtome, and 5 μm sections were stained for histological analyses. The slides were deparaffinized in toluene and rehydrated in a descending scale of ethanol baths. After antigen retrieval, the sections were blocked with 3% BSA for 30 min at room temperature or overnight at 4°C. Then, the sections were incubated with primary antibodies (summarized in Table S3) for 2 h at RT, washed in PBS and incubated for 1 h with a fluorescent secondary antibody and DAPI according to the manufacturer’s instructions. Finally, the slides were washed with PBS and mounted with ProLongTM Gold antifade reagent (Invitrogen). Histological images were analyzed via ImageJ, and three different areas were analyzed for each mouse.

Human tissue microarrays were obtained from the French Kidney Cancer Research Network UroCCR (Centre Hospitalier Universitaire de Bordeaux - Hopital Pellegrin), and IL34 protein levels were analyzed via IHC. The expression of IL34 was scored by a board-certified pathologist. Up to three different tumor areas were analyzed for each patient, and the histological score was calculated as the percentage of labeled cells (0–100%) multiplied by the staining intensity (0–3).

#### Vessel permeability miles assay

Vessel permeability was assessed via the Miles assay as previously described.^60^ Briefly, 18 days after implantation, tumor-bearing mice were anesthetized and injected intravenously with 200 μL of 1% Evan’s blue solution (Sigma). Fifteen to twenty minutes after injection, the mice were sacrificed, and the circulating Evan’s blue was removed via transcardiac perfusion with PBS. Primary tumors and contralateral kidneys were collected, weighed, and incubated in formamide for 24 h at 56°C under agitation. The absorbance of the extracted dye was quantified with a spectrophotometer at 620 nm and normalized to the tumor weight.

#### BMDM isolation and transwell migration assay

Bone marrow cells were collected from the tibia and femurs of BALB/c mice and centrifuged for 10 min at 300 × g at room temperature. The cells were subsequently resuspended in complete DMEM containing 50 ng/mL mouse M-CSF (416-ML-050/CF, Biotechne). The media was replaced every 2–3 days to remove unwanted cells, and after 8 days, the BMDMs were used for subsequent analyses. For the Transwell migration assay, 1.5 × 10^5^ renal cancer cells were seeded in complete DMEM at the bottom of each well of a 24-well plate. The next day, the media was replaced with 650 μL of fresh media. After 24 h, 1 × 10^5^ isolated BMDMs were added to 3 μm inserts and cocultured with cancer cells for an additional 24 h. Finally, migrated BMDMs were fixed in 4% formaldehyde and stained with DAPI (1:2000). After the removal of nonmigrated cells via a cotton swap, the DAPI-positive cells were imaged via a Nikon videomicroscope (9 fields/insert, 10X magnification) and counted via Fiji.

#### RNA extraction and real-time qPCR (RT‒PCR)

Total RNA was extracted via TRI Reagent (TR118-200, Euromedex) according to the manufacturer’s protocols. Then, 1–2 μg of total RNA was reverse-transcribed into cDNA via a high-capacity cDNA reverse transcription kit (Applied Biosystems, 4368814). Finally, the qPCR products were prepared via either EurobioProbe or EurobioGreen master mix (AEMMX03H or GAEMMX02H; Eurobio Scientific), and the resulting cDNA was amplified via the AriaMx Real-time PCR System (Agilent Technologies). The list of primers used is summarized in Table S4.

#### Protein extraction, Western blot analysis and ELISA

The cells were lysed via either RIPA buffer (25 mM Tris-HCl, 150 mM NaCl, 1% NP40, 0.1% SDS, 0.5% sodium deoxycholate) for Western blot (WB) analysis or TH buffer (50 mM Tris-HCl pH 7.4, 140 mM KCl, 1 mM EDTA, 1 mM EGTA, 1% Triton-X) for ELISA. Lysis buffers were supplemented with proteases and phosphatase inhibitors (Roche), and the protein concentration was determined via a BCA assay. For WB analysis, blotted PVDF membranes were incubated with Revert 700 total protein stain (cat. 926--11011, LI-COR) following the manufacturer’s instructions or with primary antibodies overnight at 4°C. Finally, the membranes were incubated with IRDye secondary antibodies diluted in Odyssey® Blocking Buffer (927--40000, LI-COR) for 1 h at room temperature and visualized via an Odyssey Imaging System (LI-COR). The list of primary antibodies used is summarized in Table S3. For ELISA quantification of secreted IL34, either 500 μg of total protein or 100 μL of mouse plasma was analyzed with a Mouse IL-34 Quantikine ELISA Kit (M3400, RD System).

#### Flow cytometry of circulating monocytes

Circulating monocytes from peripheral blood were analyzed as previously described with a few modifications.^61^ At the time of mouse sacrifice, 100 μL whole blood samples were collected in a tube containing 10 μL 0.5 M EDTA. Then, 50 μL of the sample was added to 1 mL of red cell lysis buffer (155 mM NH_4_Cl, 10 mM KHCO_3_, 0.1 mM EDTA) and incubated at RT for 10 min. The cells were then washed once and resuspended in 50 μL of 2% FBS–PBS, after which 5 μL of antibody cocktail (Table S3) was added. After 15 minutes of incubation at 4°C, the cells were washed, resuspended in 300 μL of 2% FBS–PBS and analyzed via a BD LSRFortessa flow cytometer.

#### snRNA-sequencing

Fresh samples were quickly frozen in liquid nitrogen, stored at −80°C, and processed the same day for cDNA library generation. Nuclei were isolated as previously described with minor changes.^62^ Briefly, frozen samples were homogenized in ice-cold lysis buffer (10 mM TrisHCl pH 8, 250 mM sucrose, 25 mM KCl, 5 mM MgCl2, 0.1% Triton X-100, 0.2 U/μL RNasin Plus RNase Inhibitor), strained through a 70 μm filter and collected in an Eppendorf tube. After centrifugation, the nuclei were resuspended in 1X RNase-free PBS containing 0.1% BSA and RNasin Plus RNase Inhibitor by gently pipetting 5 times via a regular-bore pipette tip. After an additional step of centrifugation, the nuclei were passed through a 20 μm strainer, stained with DAPI, counted on a hematocytometer, and finally diluted to 1,000 nuclei/μL. Subsequently, 17,000 nuclei/sample were processed on the OneCell platform (TBMCore, University of Bordeaux) for gel bead-in-emulsion (GEM) and library generation via the Chromium Next GEM Single Cell 3′ GEM, Library & Gel Bead Kit v3.1 (10X Genomics). The libraries were sequenced via an Illumina NovaSeq 6000 (GeT-PlaGe facility, Toulouse). The raw sequencing reads of the 8 libraries were aligned to the mm10 (2020-A) reference genome, and gene-count matrices were generated with 10x Genomics Cell Ranger v3.^63^ The ambient counts were removed from the matrices via CellBender,^64^ and the libraries were subsequently merged and further processed via Seurat (Seurat V3,^65^). Cells with >400 UMIs and between 200 and 6000 genes were retained. These 39,681 cells were normalized, regression was performed for the number of UMIs per cell, and the 2000 most variable genes were selected for principal component analysis on 18 principal components. Clustering was performed with the Leiden algorithm at a resolution of 0.2, and the data were visualized in a uniform manifold approximation and projection (UMAP). This resulted in 14 clusters that were annotated via canonical marker genes in 5 major cell types. The immune compartment consisting of 7,207 cells was further subclustered and annotated via the same approach with 30 principal components at a resolution of 1. Differential expression was calculated with the Wilcoxon signed-rank test implemented in Seurat.

### Quantification and statistical analysis

#### Data analysis of the TCGA-KIRC cohort

Gene expression and survival data from patients in the TCGA-KIRC cohort (*n* = 510) were analyzed via RStudio. Survival analysis was performed by separating patients into 2 groups on the basis of IL34 expression. The optimal cutoff point was determined via the surv_cutpoint function, and Kaplan‒Meier survival plots were generated via the survival and survminer packages. Gene Ontology (GO) analyses of biological process (BP) terms were performed on the 542 IL34-correlated genes. The 28 obtained GO terms were clustered into 7 clusters via the clusterProfiler and rrvgo packages. Spearman correlation coefficients (*p* < 0.05, |R|> 0.4) were utilized to identify genes coexpressed with IL34.

#### Statistical analyses

All the data presented herein were obtained from three or more biological replicates and are presented as the mean ± SEM, unless otherwise indicated, and were analyzed via RStudio version 4.2 (https://www.rproject.org/) or GraphPad Prism. Statistical tests are indicated in the figure legends. Values of *p* < 0.05 were considered statistically significant (∗*p* < 0.05; ∗∗*p* < 0.01; ∗∗∗*p* < 0.001; ∗∗∗∗*p* < 0.0001).

## Supplemental information

Document S1. Figures S1–S6

Table S1. DEG in MD_TAM (IL34-OE vs. Ctrl)

Table S2. Selected top DEG

Table S3. List of antibodies

Table S4. List of primers
