# Supplementary material for: Targeting the IL34-CSF1R axis improves metastatic renal cell carcinoma therapy outcome via immune-vascular crosstalk regulation
Source: iScience. 2025 May 26;28(6):112752. doi: 10.1016/j.isci.2025.112752 (PMC12177177; doi:10.1016/j.isci.2025.112752)
Supplement: Document S1. Figures S1–S6 [file mmc1.pdf]

iScience, Volume *28*

Supplemental information

Targeting the IL34-CSF1R axis improves metastatic renal cell carcinoma therapy outcome via immune-vascular crosstalk regulation

Andrea Emanuelli, Wilfried Souleyreau, Tiffanie Chouleur, Bram Boeckx, Yasmine Pobiedonoscew, Lindsay Cooley, Marie-Alix Derieppe, Julie Martineau, Damien Ambrosetti, Jean-Christophe Bernhard, Catherine M. Sawai, Diether Lambrechts, Thomas Mathivet, and Andreas Bikfalvi

Supplemental Figures

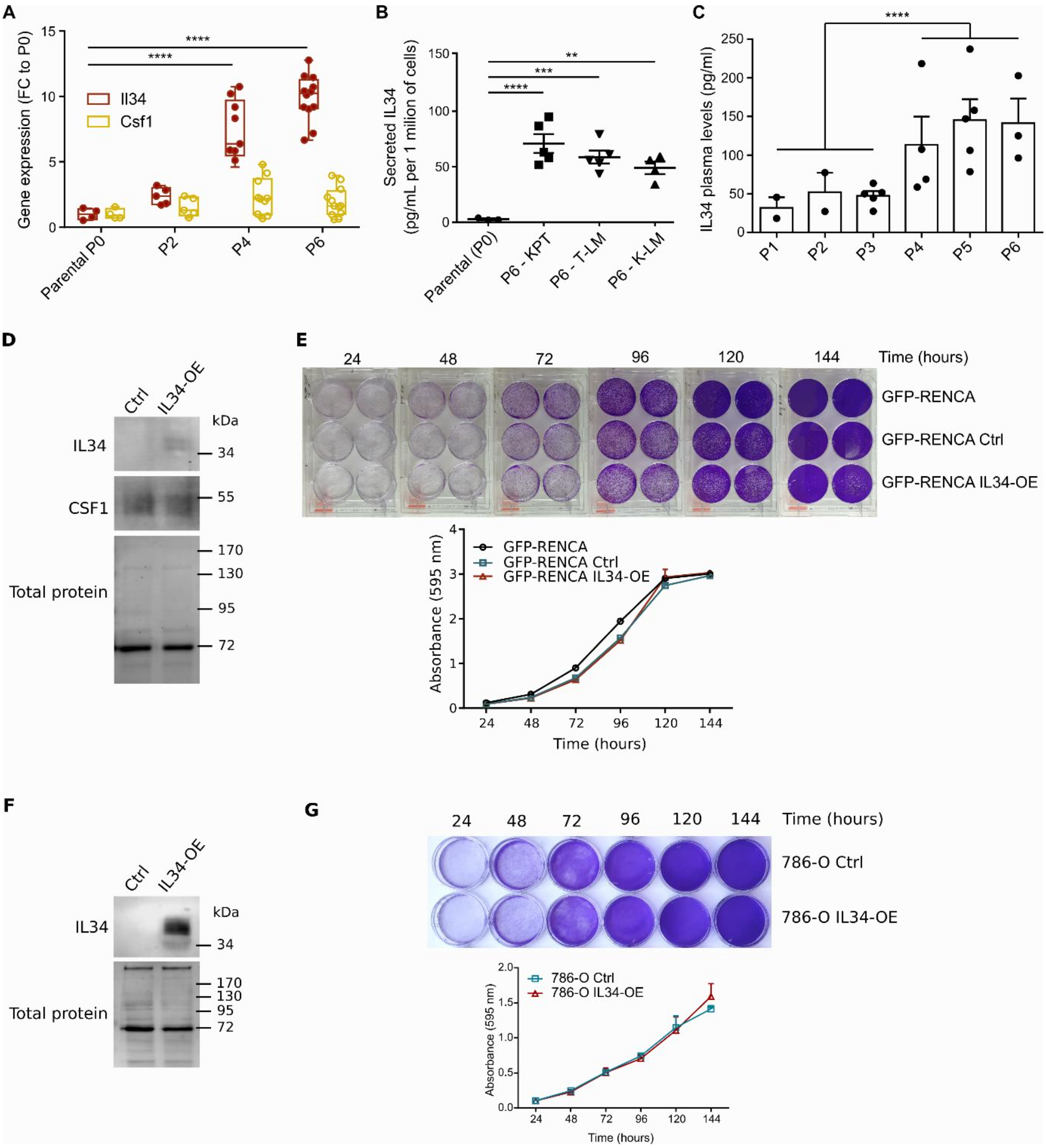

Figure S1. Renca-derived IL34 is upregulated in more aggressive mouse renal tumors and does not affect the proliferation of cancer cells

(A) Gene expression analysis (relative to the *Hprt1* gene) of *Il34* and *Csf1* in mouse Renca cells after isolation from primary renal tumors during subsequent implantation-extraction cycles. Median, min to max. Two-way ANOVA, **** p<0.0001. FC, fold change.

(B) ELISA of supernatants collected from cultured Renca cells after six passages *in vivo* and based on the implantation-extraction modality (see Cooley et al.,[7]). KPT, kidney primary tumor; K-LM, kidney-to-lung metastases; T-LM, tail-to-lung metastases. Means ± SEM. One-way ANOVA, ** p<0.01, *** p<0.001, **** p<0.0001.

(C) ELISA of blood samples collected from serially passaged tumor-bearing mice via the KPT implantation-extraction method. Mean ± SEM. Mann–Whitney U test, **** p<0.0001.

(D) Western blot analysis of supernatants from IL34-overexpressing or control Renca cells.

(E) *In vitro* proliferation assay by staining Renca with crystal violet, which revealed that IL34 did not affect cell growth (top). The absorbance of the resulting crystal violet was quantified via a spectrophotometer at 595 nm (bottom). Mean ± SEM (n=2 independent experiments).

(F) Western blot of supernatants from IL34-overexpressing or control 786-O cells.

(G) *In vitro* proliferation assay of human 786-O renal cancer cells by crystal violet staining (top). The absorbance of the resulting crystal violet was quantified via a spectrophotometer at 595 nm (bottom). Mean ± SEM (n=2 independent experiments).

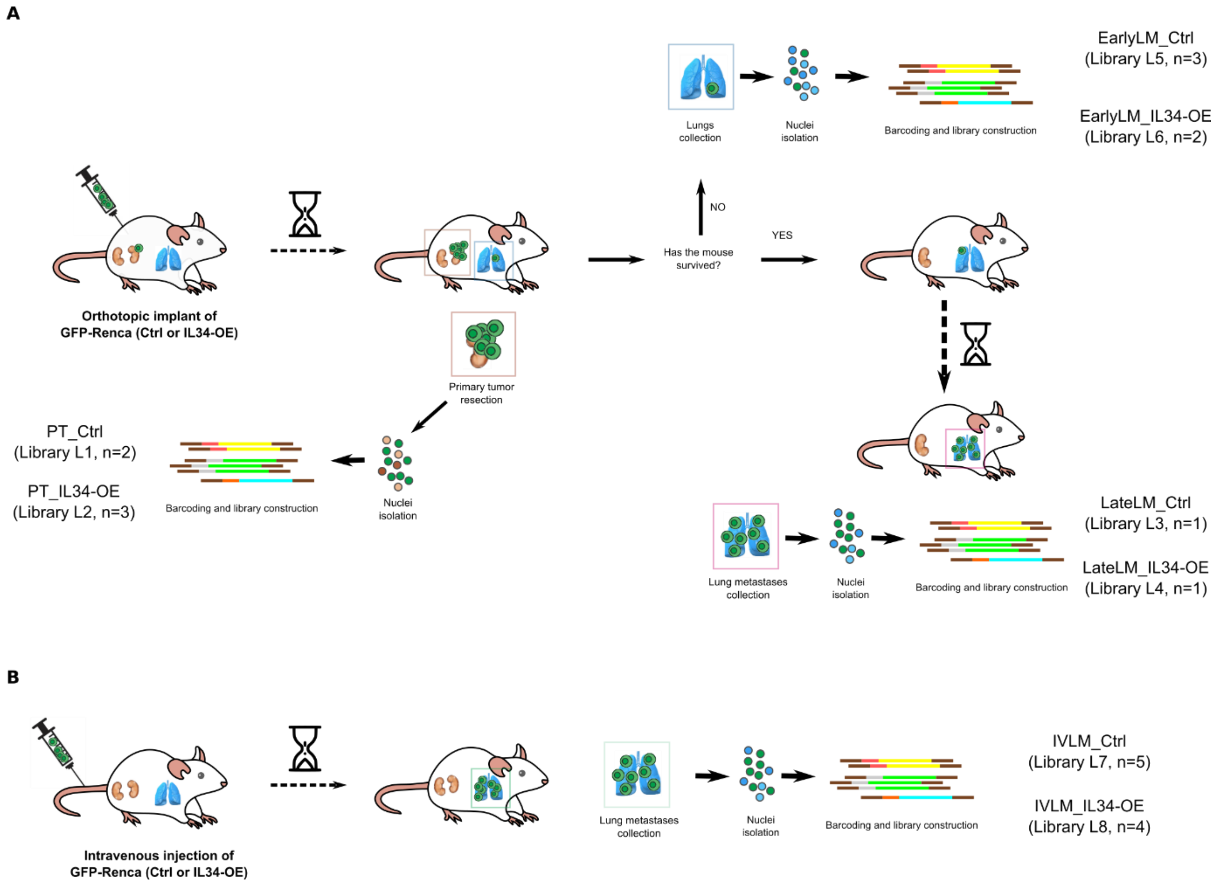

Figure S2. Workflow for sample generation and library construction for snRNA-seq

(A) and (B) Ctrl or IL34-OE Renca cells were orthotopically implanted into the left kidney of BALB/c mice to generate samples for cDNA libraries from L1 to L6 (A) or injected into the caudal vein for L7 and L8 library construction (B).

A

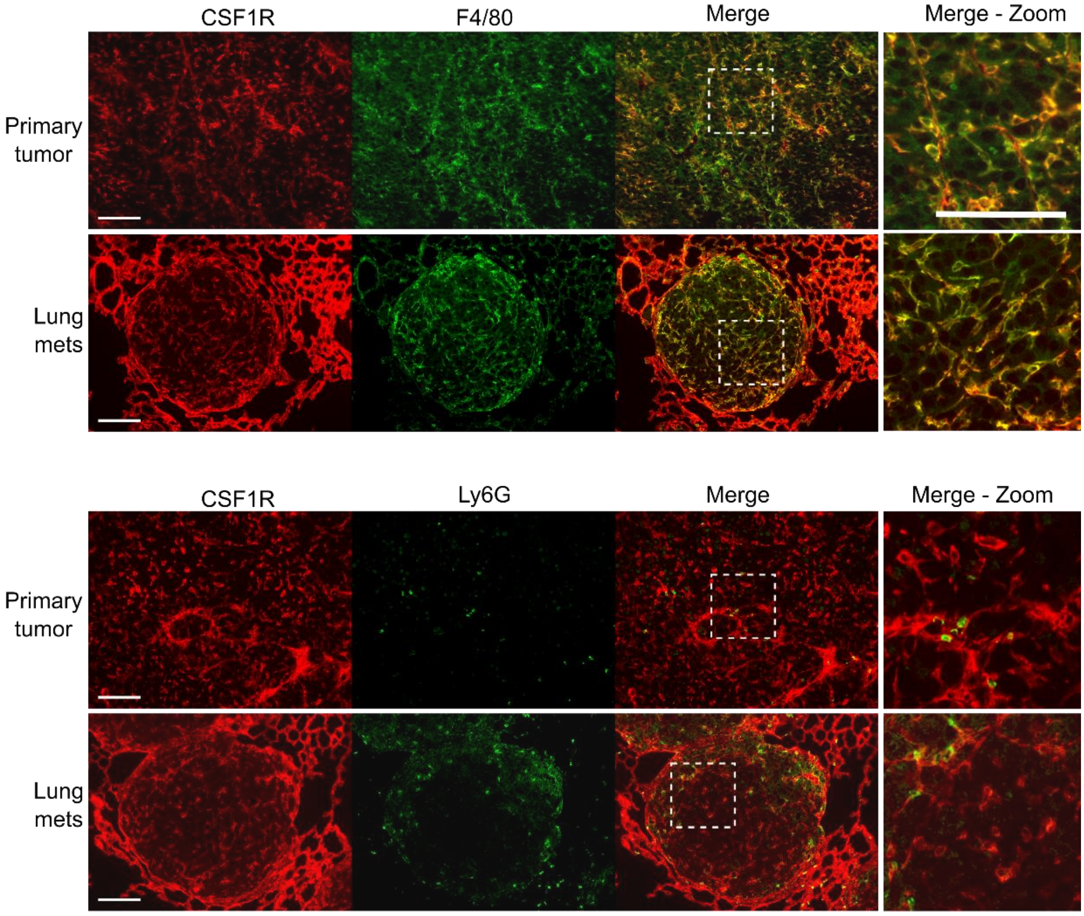

B

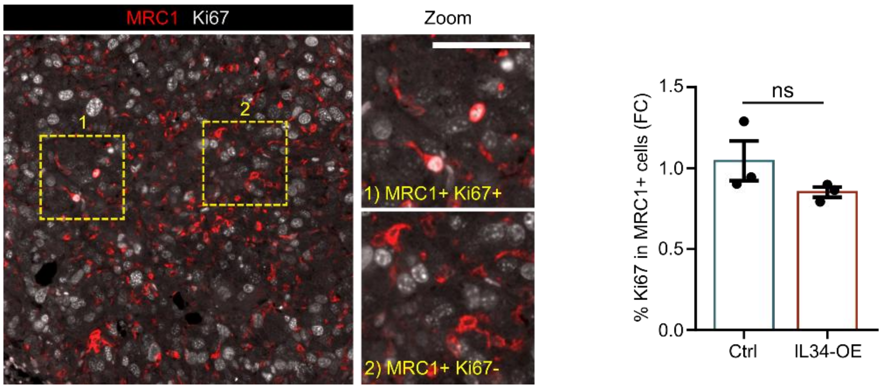

Figure S3. In the Renca mouse model, CSF1R expression was restricted to MD-TAMs

(A) Histological images of Renca-generated primary tumors or lung metastases indicating that CSF1R-expressing cells were predominantly MD-TAMs (i.e., F4/80-expressing cells). Scale bar, 100 μm.

(B) Representative image (left) and quantitative count (right) of proliferative (i.e., Ki67 positive) protumor (i.e., MRC1 positive) TAMs in lung metastases (n = 3 mice per group). Scale bar, 50 μm. Mean ± SEM. Student's t test. FC, fold change.

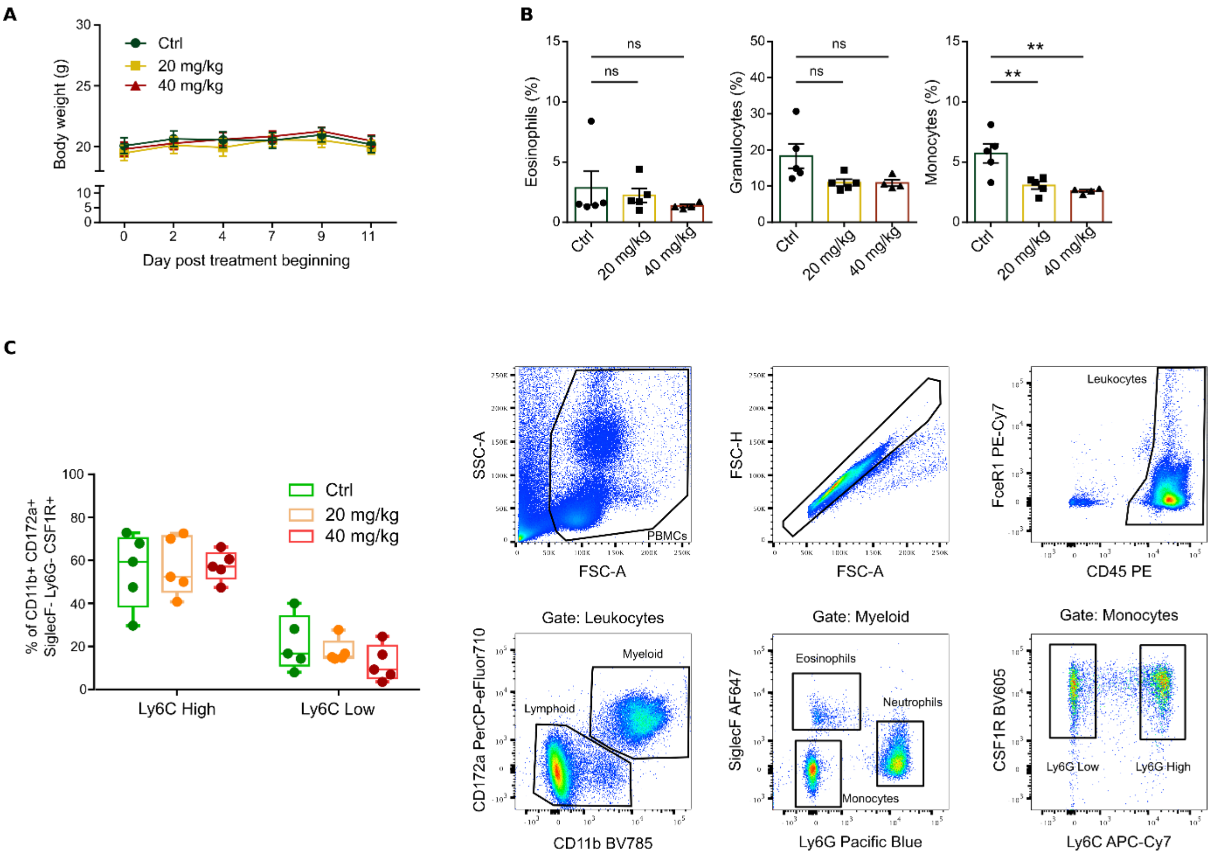

Figure S4. Minor systemic effects of pexidartinib

(A) Body weights of the mice. Mean ± SEM.

(B) Myeloid cell counts in the blood after treatment with different doses of pexidartinib (n = 5 mice per group). Mean ± SEM. One-way ANOVA, ** $p<0.01$.

(C) Left, quantification of circulating Ly6C-high or Ly6C-low monocytes in mice treated with 20 or 40 mg/kg pexidartinib compared with those in untreated mice (i.e., Ctrl). Median, min to max. Right, FACS gating strategy to identify circulating monocytes in the blood.

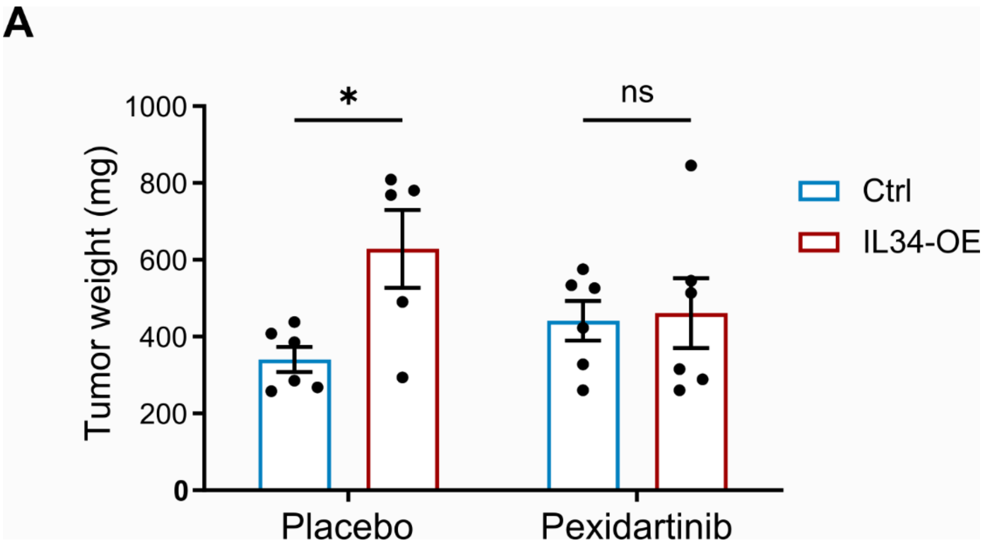

Figure S5. Weights of primary tumors in pexidartinib treated mice

(A) Renca tumors were collected for the Miles assay shown in Figure 5E, and weighed to assess their growth. Mean ± SEM. Two-way ANOVA, *p<0.05

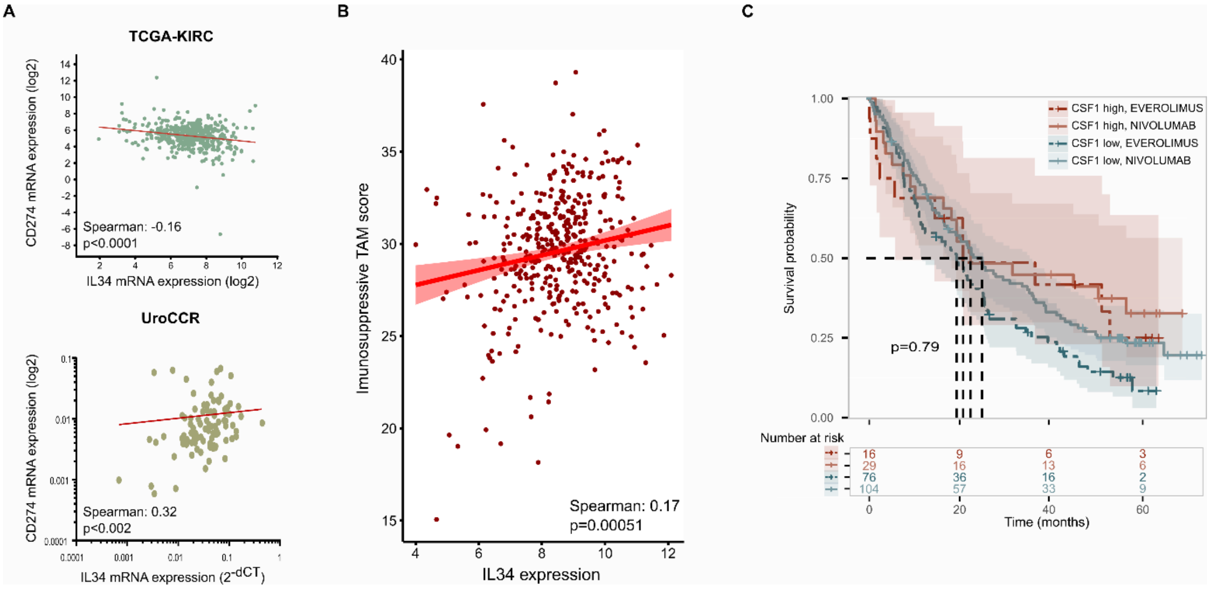

Figure S6. Correlation between IL34 expression and tumor immunosuppression in RCC patients

(A) Spearman correlation analysis of IL34 expression with CD274 expression in the KIRC-TCGA (top) and UroCCR (bottom) databases.

(B) Spearman correlation analysis of IL34 expression with gene signature of immunosuppressive TAMs (i.e. CD38+MSR1+MRC1-) in the KIRC-TCGA database.

(C) Kaplan–Meier survival analysis of patients stratified on the basis of CSF1 expression (high vs low) who received treatment (everolimus vs nivolumab, CheckMate CM-025 cohort).
